# Description of Placement Procedures for Common Methods Used in Equine Emergency Rescue Using a Simplified Loops System

**DOI:** 10.3390/ani9080529

**Published:** 2019-08-05

**Authors:** John Madigan, Lais Costa, Samantha Nieves, Molly Horgan, Kirsten Weberg, Monica Aleman

**Affiliations:** Department of Medicine and Epidemiology, School of Veterinary Medicine, University of California, Davis, CA 95616, USA

**Keywords:** technical rescue, recumbent, equids, welfare, stranded

## Abstract

**Simple Summary:**

Horses can become entrapped, stranded in confined spaces, exhausted, or injured and become recumbent and unable to rise. Horse can injure themselves struggling to rise and endanger the personnel trying to rescue them. Successful rescuing of these animals often requires expedient manipulations. We designed a lightweight portable kit for movement or lifting of a recumbent equine using a novel Loops System. Using an equine life size mannequin, we describe the placement procedures of the Loops System without knots, J hooks or specialized accessory devices. Five maneuvers commonly used in equine technical rescue are illustrated with step-by-step instructions for forward assist, rear assist, full body roll, rear drag, and vertical lift.

**Abstract:**

Entrapped, stranded and recumbent equids often require emergency rescue. The success of the rescue is often affected by secondary injuries from struggling of the horse to rise and from injury secondary to attempted rescue by pulling on the head or limbs of the equid. Therefore, having ready access to simplified rescue equipment which can be easily applied would be desirable. The devices currently available for these manipulations are not always readily available at the site of an incident. Here, we describe and illustrate the step-by-step use of a Loops System consisting of 183 cm round slings, which can be positioned on the recumbent horse utilizing commercially available and reasonably priced equipment. The Loops System is basically composed of four round slings placed in such a way that utilizes the skeletal system for support. The procedures are illustrated utilizing a recumbent life-size horse model or mannequin. We suggest that the Loops System kit may allow enhanced ability for responders to provide care to a recumbent horse.

## 1. Introduction

Equids can become trapped in confined spaces, including horse trailers, ditches, ravines, tree forks, mud, wells, and damaged or improper fencing. Incidents such as hurricanes, earthquakes and floods can lead to need for extraction from confined spaces. Also, horses with extreme weakness following an injury or fall, or with a history of neurologic or musculoskeletal diseases, can be unable to stand, making transport and even survival difficult [1,2,3]. Veterinarians may need to lift a recumbent equine to perform an evaluation and make a diagnosis and prognosis. Additionally, tactical large animal rescue is a field that has evolved to aid animals in these situations [4]. Because of the hazards of the size of the animals and the behavior of equids, it is critical that those attempting to work with recumbent or stranded equines have proper training and immediate access to equipment to aid in the rescue process. Furthermore, due to the nature of equids as prey animals, the recumbent or stranded equid often struggles incessantly, making it difficult for responders to provide aid. Prolonged recumbency of horses is associated with secondary injuries, including cranial trauma, eye injuries, myopathies, musculoskeletal injury, and nerve paralysis [5].

Effective sling or lifting devices used in veterinary medicine for large animals utilize the core skeletal system for support due to the considerable weight of these animals [6]. Veterinary hospitals use specialized slings for compromised equines, but these devices are relatively expensive ($2000–$5000 USD) and not widely available in many field settings [6,7]. Therefore, we designed a simplified, less-expensive (estimated cost under $350 USD), easy to apply, rescue system for use only in the short-term movement or brief lifting of the equine rescue patient to allow the horse to be moved to a safer location or allowed to try to stand. 

The loops’ round sling system referred to as the Loops System utilizes skeletal system support similar to the UC Davis Large Animal Lift and in humans, full body safety harnesses [8,9]. The Loops System is highly portable, fits in a small duffel bag (9 kg weight and 30 cm × 54 cm × 24 cm dimensions), simple to apply with written step-by-step directions, does not require knots or hooks, and can be placed rapidly from a safe area behind the back of the horse away from the limbs. The ultimate goal of describing the use of the Loop System is to improve equine welfare.

Here we describe the use of the Loops System for assisting rescue of recumbent equids by describing placement for five maneuvers commonly used in equine technical rescue-forward assist, rear assist, roll, rear drag, and vertical lift, utilizing a recumbent life-size horse mannequin model. 

## 2. Materials and Methods 

### 2.1. Loops System 

**Figure 1:**
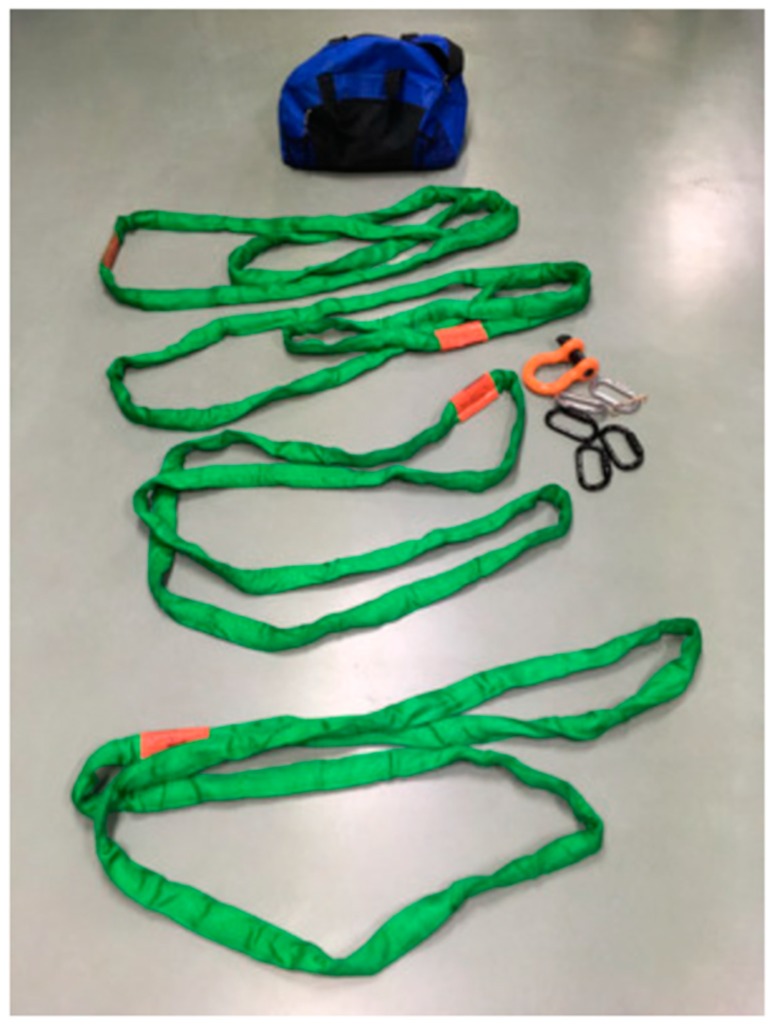
Loops System.

This Loops System used in this study consists of four Lift-All Tuflex Green EN60 183 cm, Roundsling [10], four Petzl^®®®^ OK screw lock carabiners, one 2.2 cm × 11.4 cm bolt-type D-ring anchor shackle with a 1.9 cm × 8.25 cm pin (Figure 1). Each of the Lift-All Tuflex Green EN60 round sling has a rated capacity range greater than 1905 kg. All items are readily available via internet order [10] or can be obtained in complete kit form (for information on kit ordering see [11]) (Figure 1).

### 2.2. Procedure for Placement of Loops System on a Recumbent Horse Model

A life-size equine fiberglass mannequin model (height at withers 170 cm, length 270 cm) in lateral recumbency was used in place of live animals, and two Lift-All EN60 X 183 cm Green Tuflex polyester round slings were used for the manipulation procedures. The vertical lift procedures have been performed in live horses [12].

**Figure 2:**
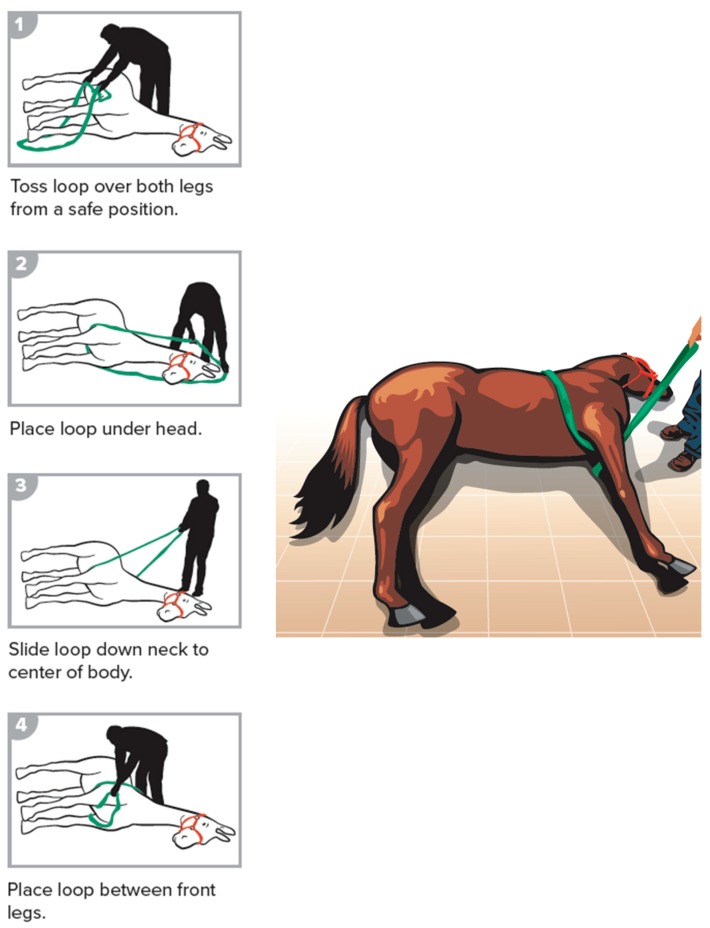
Forward assist.

**Figure 3:**
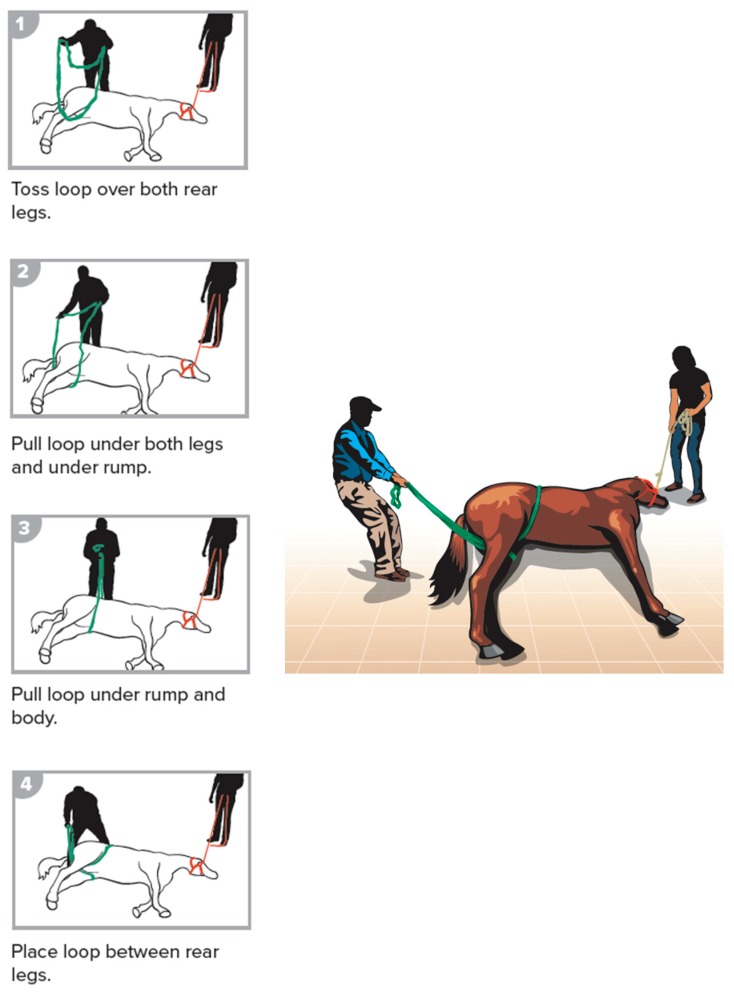
Rear assist.

**Figure 4:**
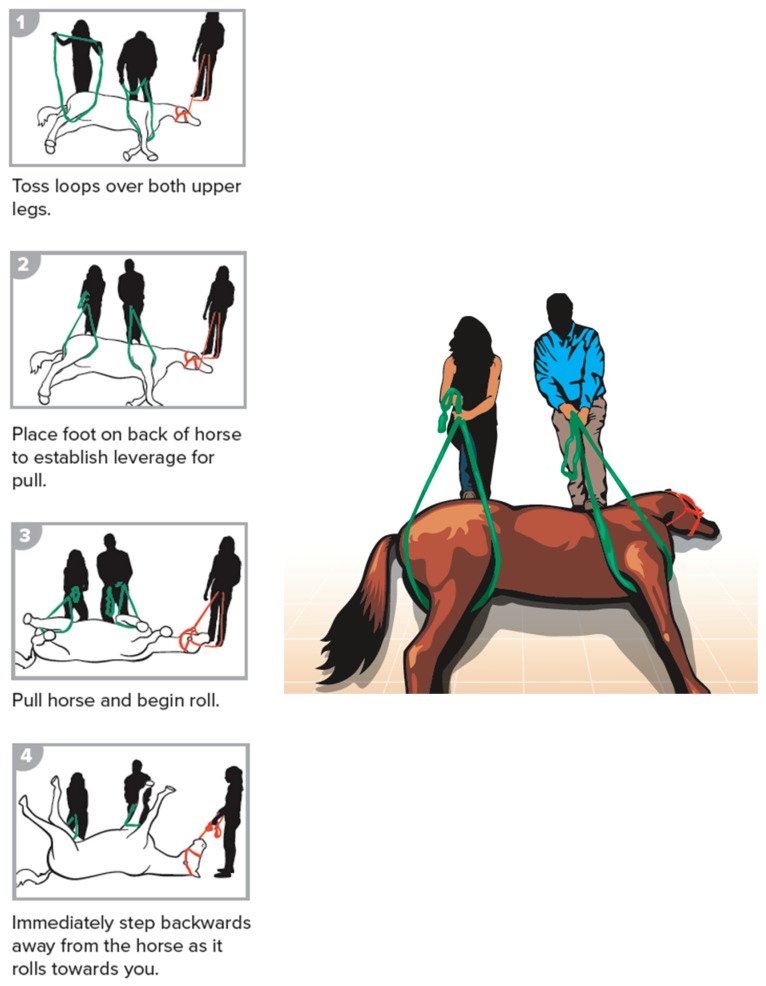
Roll of a recumbent horse.

**Figure 5:**
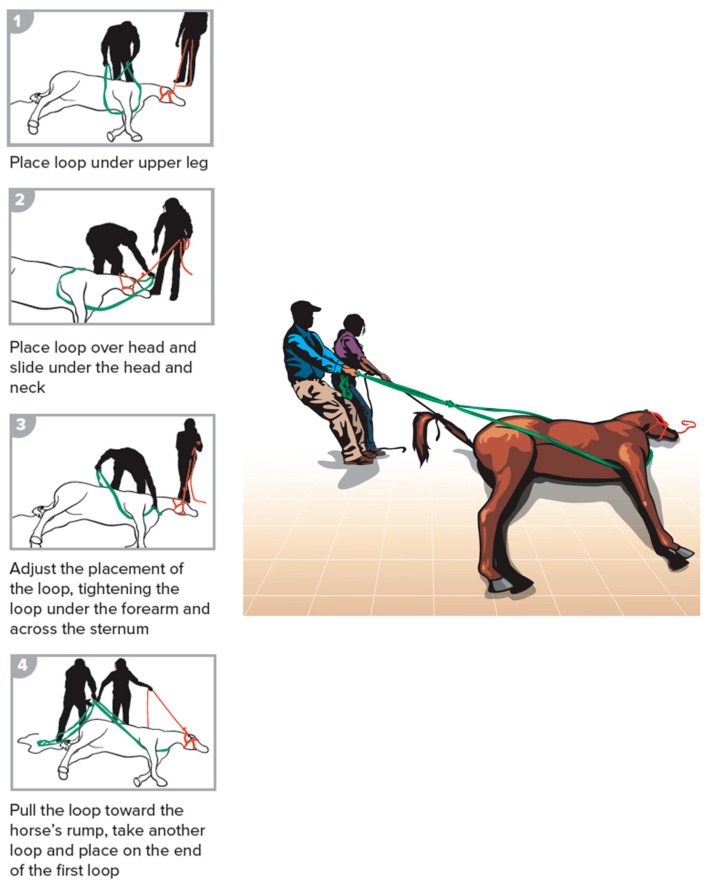
Rear drag.

**Figure 6:**
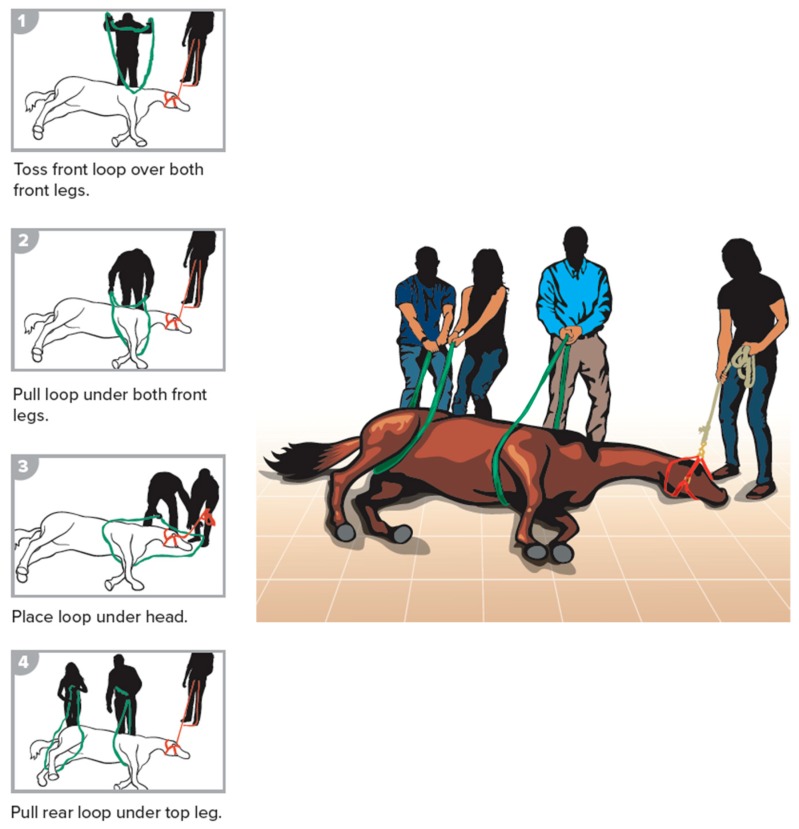
Horizontal drag.

**Figure A1:**
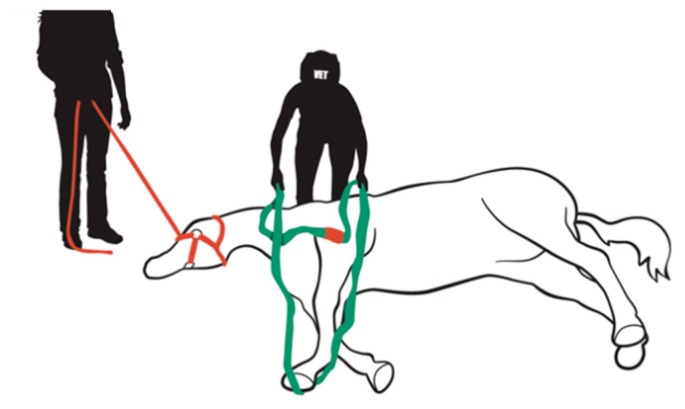
Toss the first loop over the upper front limb.

**Figure A2:**
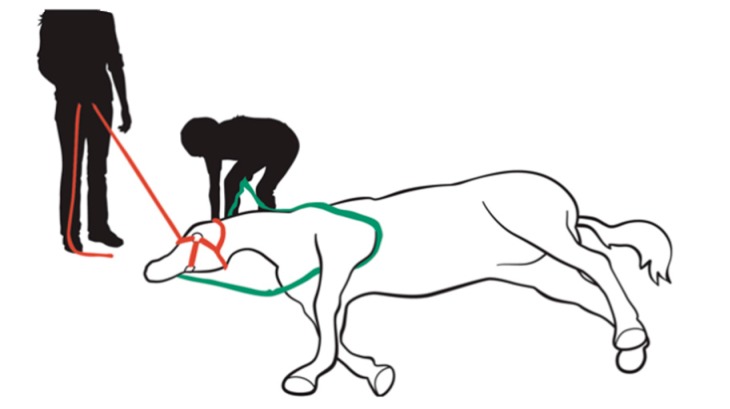
Pull the first loop towards you, and slide it under the horse’s head and neck.

**Figure A3:**
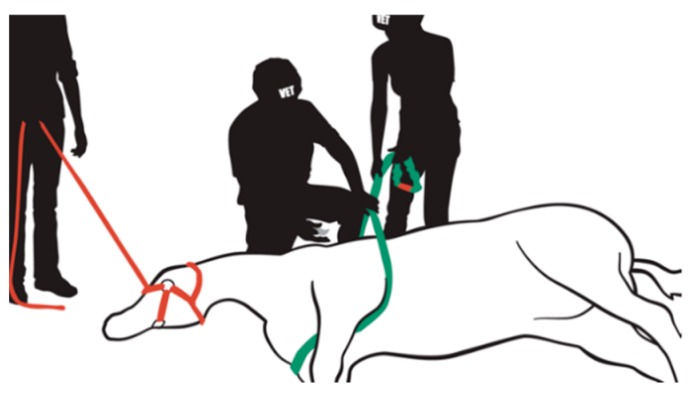
Place a piece of duct tape to temporarily secure the loop in place.

**Figure A4:**
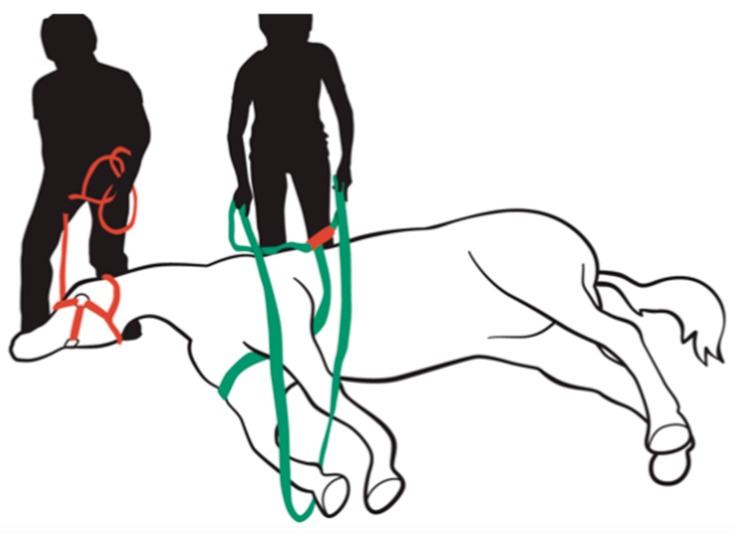
Toss the second loop over the lower front limb.

**Figure A5:**
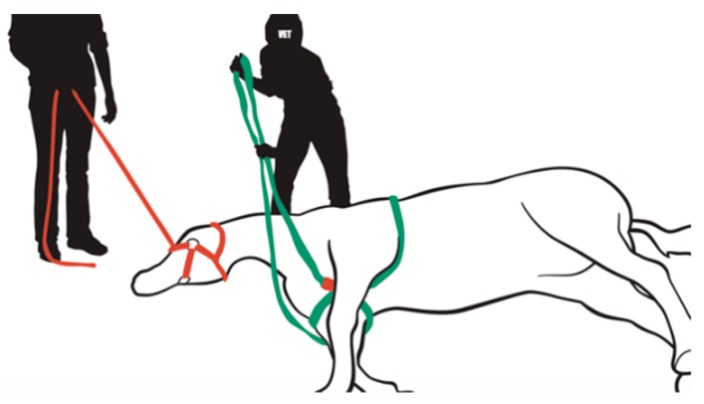
Pull the second loop towards you.

**Figure A6:**
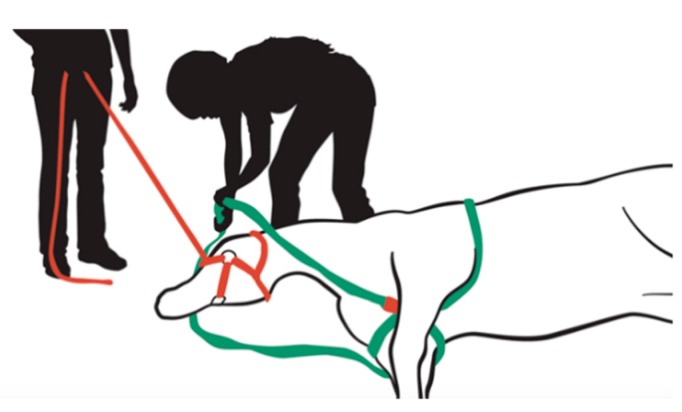
Slide the second loop under the head and neck.

**Figure A7:**
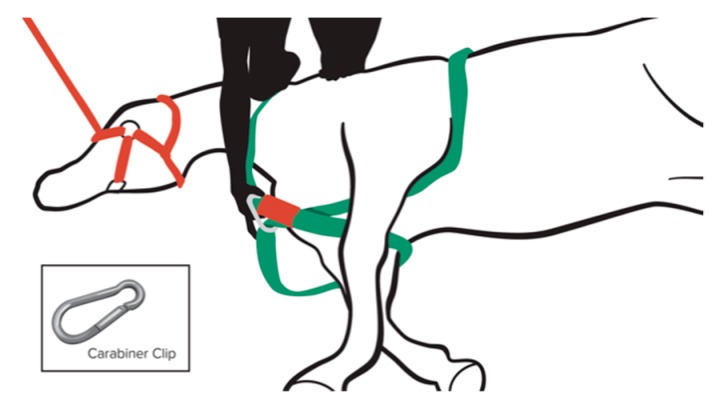
Place one carabiner in front of the sternum.

**Figure A8:**
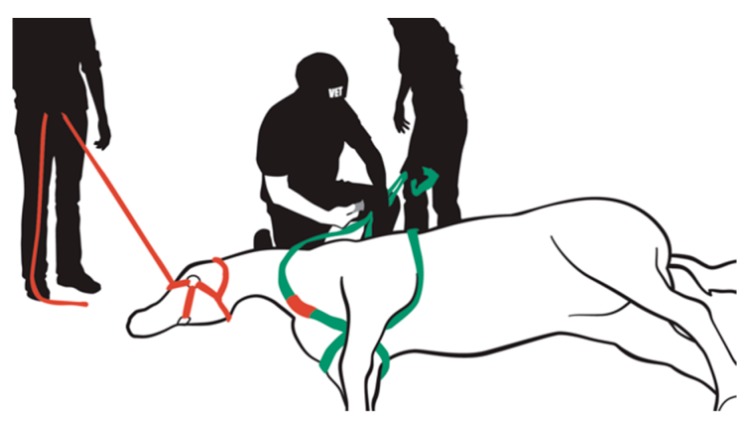
Place a piece of duct tape to temporarily secure the loop in place.

**Figure A9:**
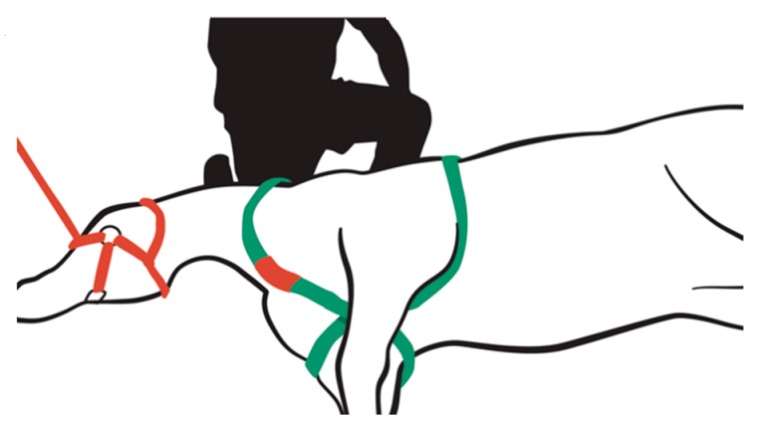
Note how the loops crisscross between the front limbs.

**Figure A10:**
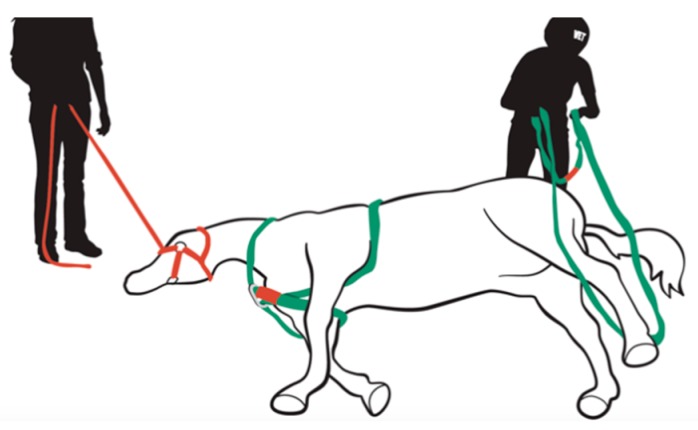
Toss the third loop over the upper hind limb.

**Figure A11:**
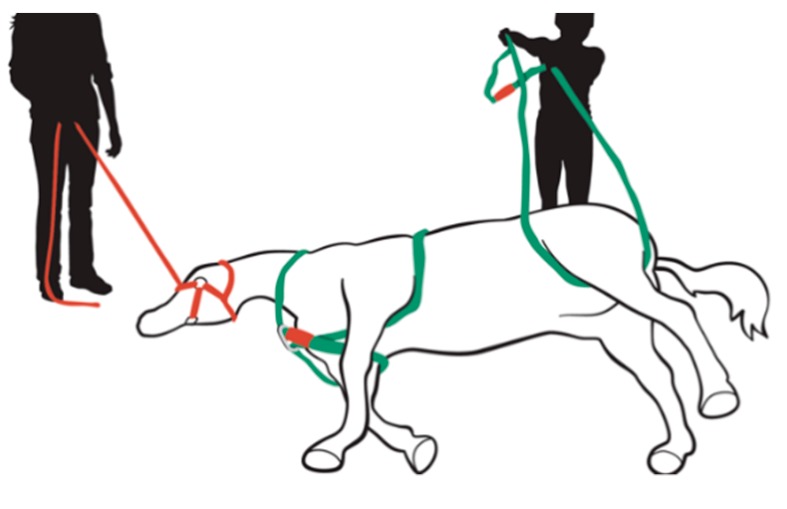
Pull the third loop towards the horse so it lays between the hind limbs.

**Figure A12:**
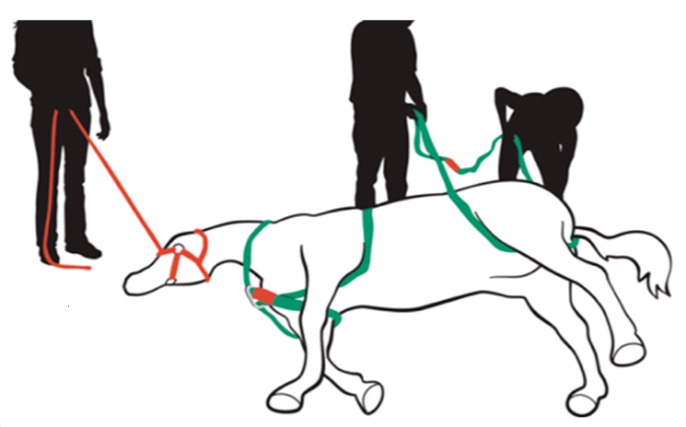
Pull the loop underneath the horse’s tail such that it crosses over the horse’s rear.

**Figure A13:**
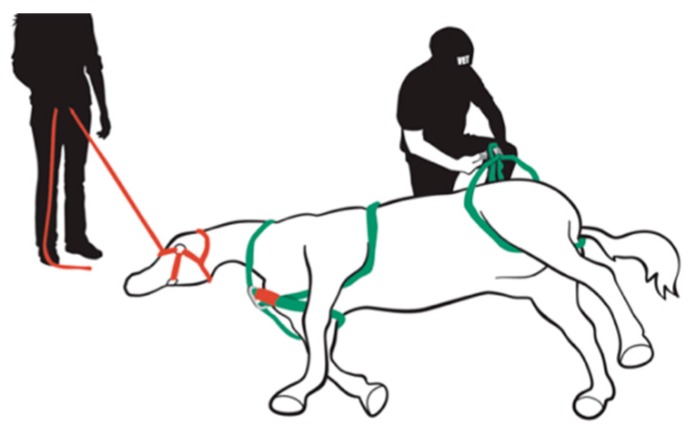
Place a piece of duct tape to temporarily secure third loop in place.

**Figure A14:**
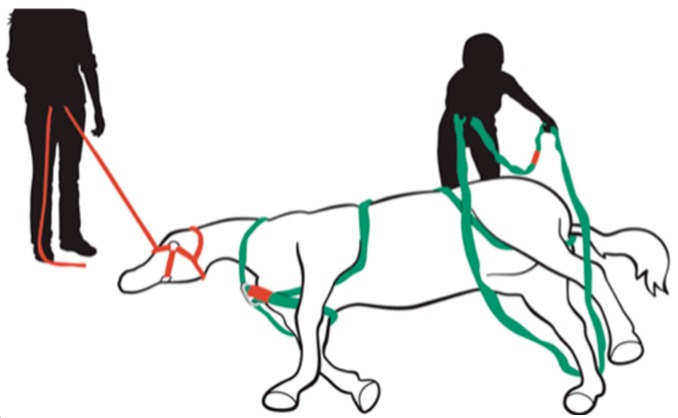
Toss the fourth loop over the lower hind limb.

**Figure A15:**
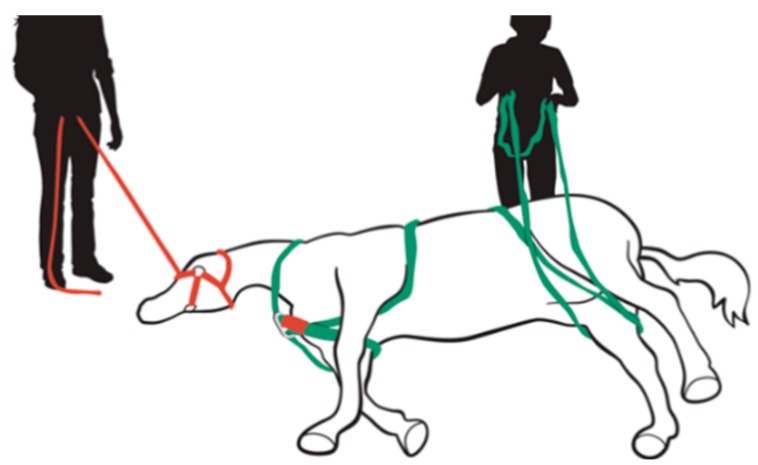
Pull the 4th loop towards the horse, such that the loop is between the hind limbs.

**Figure A16:**
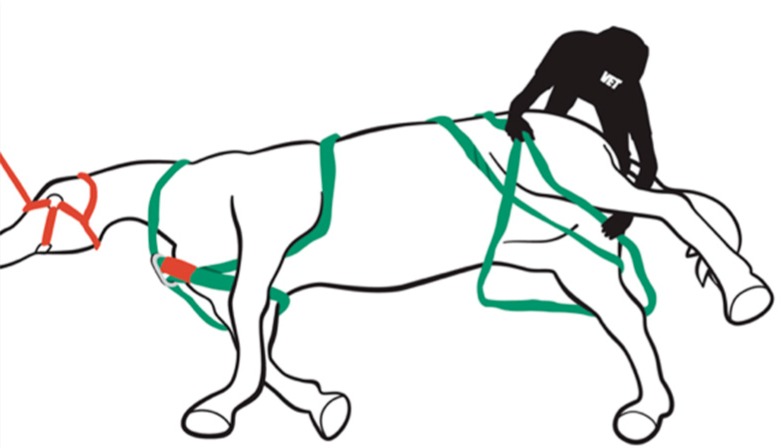
Reach and pull the fourth loop towards the rear.

**Figure A17:**
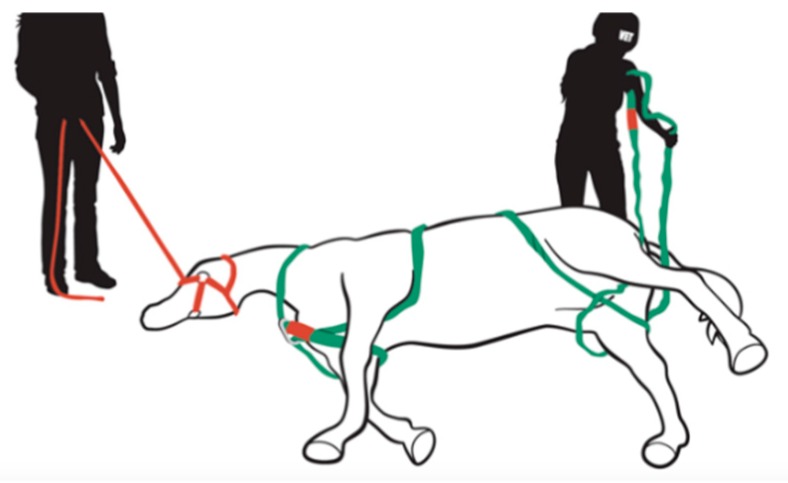
Pull the fourth loop towards the tail.

**Figure A18:**
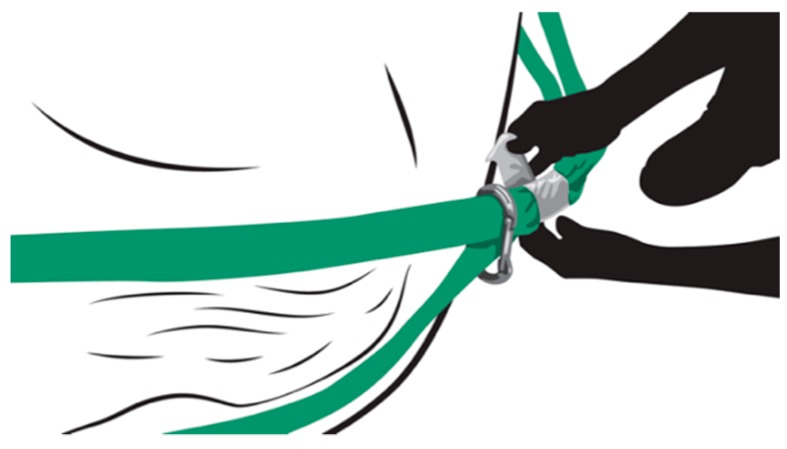
Place a carabiner between the loops going over the rump, and a piece of duct tape to secure temporarily the loops in place.

**Figure A19:**
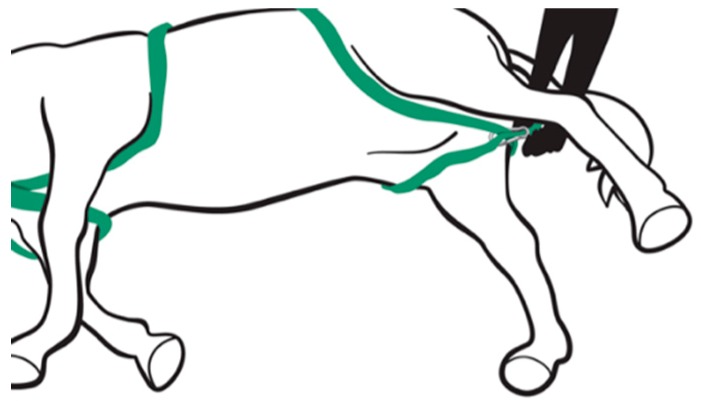
Place a carabineer between the loops crossing between the hind limbs.

**Figure A20:**
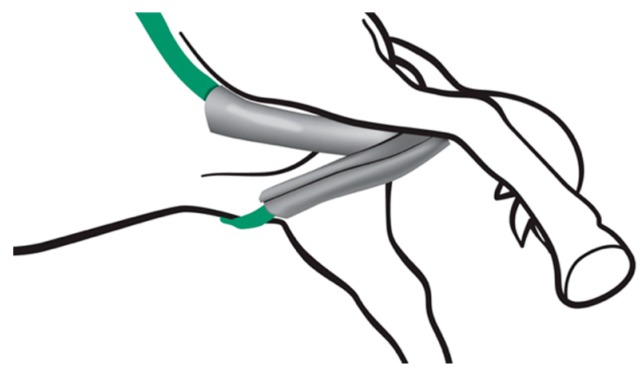
Place the clamp-on foam around each of the loops between the hind limbs.

**Figure A21:**
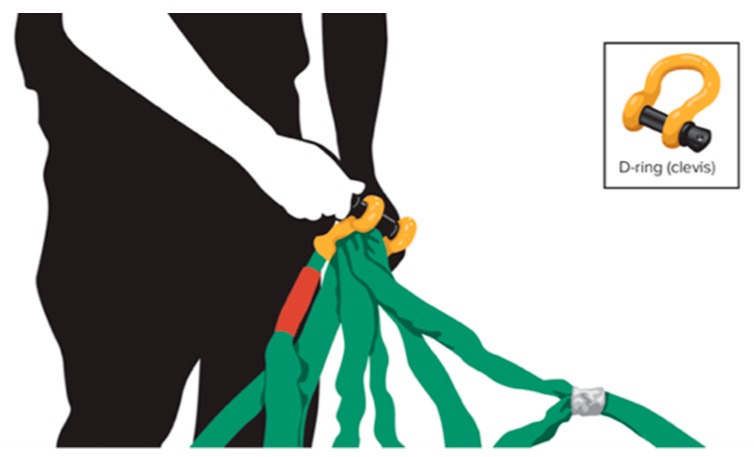
Place all four loops onto the clevis.

**Figure A22:**
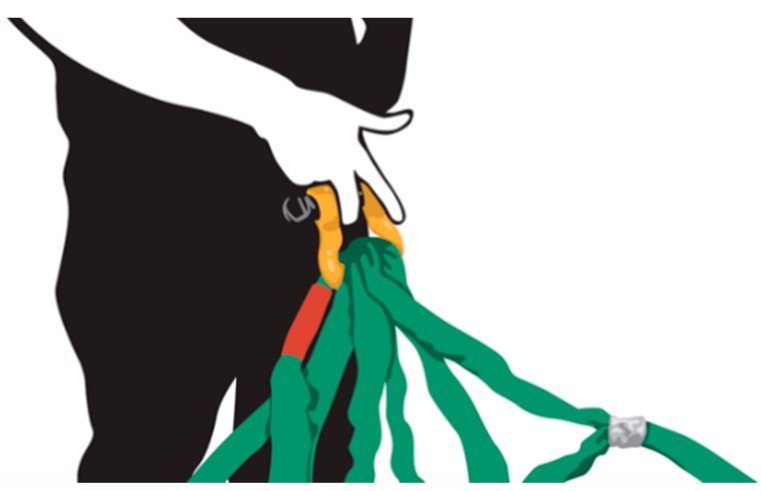
Note the arrangement of the loops on the curved part of the D-ring (clevis).

**Figure A23:**
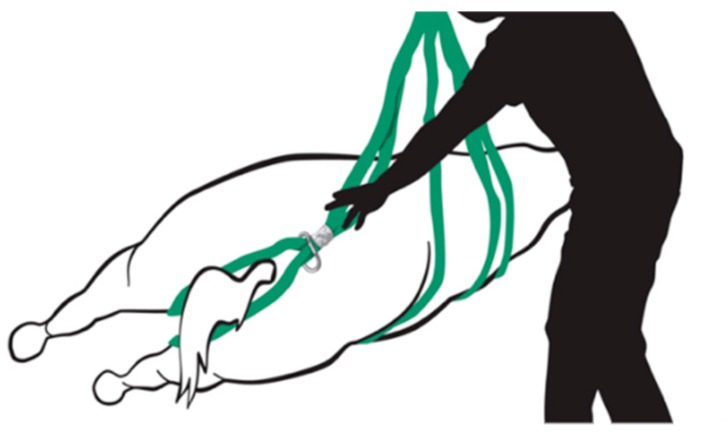
Begin lifting.

**Figure A24:**
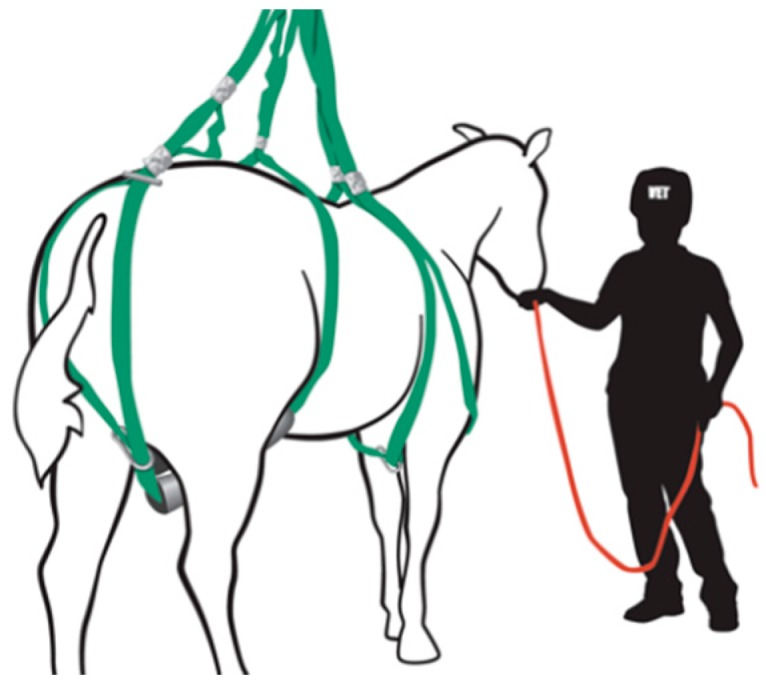
Horse on its feet.

The steps of the methods are illustrated and described below. The procedures included having an assistant reading the steps for the placement of the loops. All participating individuals (the authors) exercised safe handling techniques and stayed behind the horses’ body, away from the limbs, to mimic the live animal situation for safe standing position. Participants wore helmets and gloves (not shown in illustrations). The danger zone was defined as a semi-circle area from model’s head, around the feet, to the tail with a three-foot safety radius. The procedures included forward assist (Figure 2), rear assist (Figure 3), roll of a recumbent horse (Figure 4), rear drag (Figure 5), horizontal drag (Figure 6), and vertical lift (Appendix A Figure A1, Figure A2, Figure A3, Figure A4, Figure A5, Figure A6, Figure A7, Figure A8, Figure A9, Figure A10, Figure A11, Figure A12, Figure A13, Figure A14, Figure A15, Figure A16, Figure A17, Figure A18, Figure A19, Figure A20, Figure A21, Figure A22, Figure A23 and Figure A24). Not shown in all the illustrations is the placement of a person near the head of the horse, a head bumper for protection, and use of a skid to facilitate the movement of a recumbent horse.

## 3. Results

The Loops System was slid under the mannequin and placed in position within approximately three min for the forward assist, rear assist, rear drag, and roll procedures. Placement of the four loops for the vertical lift was not timed but takes longer than the other procedures and is illustrated in a step-by-step manner below. 

## 4. Discussion

Here, we described a novel compact system for the manipulation of the recumbent or stranded equid, using an affordable and portable device to be used in aiding weak horses and equine tactical large animal rescue. The significant advantages of this novel rescue system are: (1) It provides support by utilizing the skeletal system for safe manipulation of large animals, (2) its placement is rapid and accompanied by step-by-step directions, (3) it is simple, not requiring J hooks or knots, (4) it is very compact and portable, and (5) it employs commercially available, and reasonably priced equipment. The accessibility of this new system may facilitate the rescue of entrapped, stranded, and recumbent equids, thus having a significant impact on animal welfare.

The placement of the Loops System was directed and done with one person reading the illustrated instructions to the person applying the loops. We suggest this may aid those performing technical rescues in difficult circumstances. The application of the Loops System may provide a compact tool for first responders, veterinarians, and competent horse owners, thus greatly facilitating the success of the technical large animal rescue. Ready access to rescue equipment when a horse is trapped and struggling will potentially minimize secondary injuries as well as lessen exhaustion in the trapped equid. This system can be stored in a small duffle bag, making it convenient to have in stables or first responder vehicles. We believe the components are affordable (we estimate the Loops System components are less than $350 USD at this time) compared to other alternatives. Many excellent support systems are found in veterinary hospitals and in some communities where technical rescue teams are part of emergency response. Unfortunately, equine incidents often occur away from such prepared teams. 

Instructions listed here could be printed and placed in the bag, so the steps in placement can be followed quickly. Any use of this, or other rescue systems should be preceded by individuals being trained in safety measures for equid technical rescue. Injury to people is a substantial risk in equine technical rescue. The use of protective equipment such as helmets and gloves and prior training in safe approaches is essential to prevent serious injury to people. Future studies are recommended to evaluate the field use of the Loops System in the rescue of entrapped, stranded, or recumbent equids. 

Limitations of the study: Any studies involving mannequins instead of live horses have limitations with regard to safety, animal tolerance to support systems, and unpredictable animal movements. The personnel performing the placement of the loops here are members of the Veterinary Emergency Response Team and may have inherent skills that allow faster placement than a person who has attended a single training and then uses the loops in an animal setting. However, the presence of illustrated guidelines that can be read while performing the placement and the lack of the need for J hooks and knots suggests that this placement would proceed relatively quickly but not as fast as estimated in this brief study utilizing a mannequin. Live horses present more difficulties, and hence, the placement time would be expected to be longer in real emergency settings. Additionally, there is no quick release of the current Loops System. The authors have used the procedures and loops system in three live animal field settings: (1) forward assist to move a recumbent 300 kg body weight donkey into a horse trailer, (2) rear assist to drag a 500 kg body weight mare with dystocia from a horse trailer, and (3) lifting a 150 kg body weight recumbent sow with extensive burns.

## 5. Conclusions

The basic methodology for approaches for movement of a recumbent horse, and likely a donkey, or mule using a simplified rescue Loops System is illustrated following placement on an equine mannequin. The steps of forward assist, rear assist, rear drag and rolling the recumbent horse have been used previously in equine technical rescue but involve more cumbersome, more complex, less affordable and often less readily available equipment close to an animal incident. The use of the Loops System for vertical lift is more complex and has been tested in live horses [12]. Specific training before use is required to prevent serious injury or death to those working with a stranded or compromised equine [13].

## Appendix A

Loops System for vertical lift placement steps, Figure A1, Figure A2, Figure A3, Figure A4, Figure A5, Figure A6, Figure A7, Figure A8, Figure A9, Figure A10, Figure A11, Figure A12, Figure A13, Figure A14, Figure A15, Figure A16, Figure A17, Figure A18, Figure A19, Figure A20, Figure A21, Figure A22, Figure A23 and Figure A24.

.
